# Gut-brain axis modulation in remote rehabilitation of Parkinson’s disease: reconstructing the fecal metabolome and nigral network connectivity

**DOI:** 10.3389/fneur.2025.1644490

**Published:** 2025-08-15

**Authors:** Yuting Jin, Huan Wang, Jinan Song

**Affiliations:** Department of Rehabilitation, Shengjing Hospital of China Medical University, Shenyang, China

**Keywords:** Parkinson’s disease, gut-brain axis, short-chain fatty acids (SCFAs), α-synuclein, neuroinflammation, remote rehabilitation, vagus nerve stimulation

## Abstract

The pathogenesis of Parkinson’s disease (PD) is gradually evolving from a central neurodegeneration-centered concept to a multi-pathway pathological model at the gut-brain system level. Studies have shown that PD patients commonly exhibit dysbiosis, reduced short-chain fatty acids (SCFAs; microbial fermentation products of dietary fiber that play key roles in host metabolism and immune regulation), abnormal tryptophan metabolism, and impaired gut barrier function. These alterations may contribute to dopaminergic neuronal damage through mechanisms including neuroinflammation, oxidative stress, and α-synuclein (α-syn) aggregation. The vagus nerve plays a critical role in bidirectional gut-brain signaling, and its dysfunction may represent a key route for pathological protein transmission from the periphery to the brain. In response, remote rehabilitation and gut-targeted interventions—including probiotics, prebiotics, dietary modulation, fecal microbiota transplantation (FMT), and transcutaneous vagus nerve stimulation (tVNS)—have shown potential in improving neurological function and inflammation in both animal and clinical studies. Multimodal data analyses have revealed significant associations between SCFA levels in fecal metabolomics and brain imaging features. Despite ongoing challenges in mechanistic extrapolation, biomarker sensitivity, and translational implementation, the integration of metagenomics, metabolomics, neuroimaging, and digital therapeutics—collectively referred to as multi-omics and digital profiling techniques—represents an emerging research direction with the potential to inform future clinical paradigms for precision remote management of PD.

## 1 Introduction

Parkinson’s disease (PD) is a neurological disorder characterized by the degenerative loss of dopaminergic neurons in the substantia nigra pars compacta (SNc) and abnormal aggregation of α-synuclein (α-syn) (1–3). Pathological deposition of α-syn disrupts mitochondrial function and induces endoplasmic reticulum stress responses, thereby driving neuronal apoptosis (4, 5). This process is closely linked to the core motor symptoms of PD, essentially resulting from the imbalance of the basal ganglia-thalamocortical loop caused by the loss of nigrostriatal dopaminergic projections (6). Notably, non-motor symptoms often precede motor dysfunction, suggesting that PD pathology may originate in the peripheral nervous system (7, 8). Postmortem studies have shown that α-syn pathology initially localizes in the enteric nervous system (ENS) and subsequently propagates to the midbrain via the vagus nerve (9). This phenomenon is supported by epidemiological findings indicating that vagotomy reduces the risk of developing PD by 40% (10).

In recent years, regulatory mechanisms of the gut-brain axis—the bidirectional communication network between the gastrointestinal system and the central nervous system—have offered a novel perspective for PD research (11–13). Clinical cohort analyses reveal that up to 80% of PD patients experience gastrointestinal dysfunction 10–20 years before the onset of motor symptoms (14), and their fecal microbiota exhibit a marked decrease in Prevotellaceae and overgrowth of Enterobacteriaceae (15). This dysbiosis may result in dual pathological effects: on one hand, decreased synthesis of SCFAs compromises their regulatory capacity on microglial M2 polarization, exacerbating neuroinflammation mediated by the TLR4/NF-κB pathway and increasing proinflammatory cytokines such as IL-1β and TNF-α (16); on the other hand, enhanced intestinal permeability facilitates the translocation of lipopolysaccharide (LPS) into circulation, which activates α-syn fibrillization and impairs the blood–brain barrier, accelerating the central propagation of pathological proteins (17). Animal experiments further confirm that intragastrointestinal injection of α-syn fibrils can be transported retrogradely to the substantia nigra via the vagus nerve, inducing selective loss of dopaminergic neurons (18). These lines of evidence collectively suggest that disruption of the gut-brain axis is not only an early event in PD pathogenesis but may also serve as a critical target for therapeutic intervention.

## 2 Molecular and neural mechanisms of gut-brain axis modulation

### 2.1 Signal transmission of gut microbiota metabolites

Metabolites of the gut microbiota are involved in the central pathological processes of PD through multiple pathways, particularly playing important roles in regulating neuroinflammation, synaptic function, and oxidative stress (19). Short-chain fatty acids (SCFAs), the major products of microbial fermentation of dietary fiber, play a key role in modulating central nervous inflammation (20, 21). Butyrate promotes microglial polarization toward an anti-inflammatory phenotype (M2 type) by inhibiting histone deacetylases (HDACs), thereby downregulating the activity of the Toll-like receptor 4 (TLR4)/nuclear factor-κB (NF-κB) signaling pathway (22). Clinical studies have shown that butyrate concentration in the feces of PD patients is significantly negatively correlated with IL-6 levels in cerebrospinal fluid (23). Propionate activates free fatty acid receptor 2 (FFAR2) on enterochromaffin cells, promoting the synthesis of serotonin (5-HT), thereby regulating the synaptic plasticity of nigrostriatal dopaminergic neurons (24). Notably, the effect of propionate on dopamine metabolism is dose-dependent: low concentrations of propionate (<1 mM) enhance the activity of tyrosine hydroxylase (TH), while high concentrations (>5 mM) induce neuronal apoptosis through the accumulation of mitochondrial reactive oxygen species (mtROS) (25).

The bidirectional regulation of tryptophan metabolism further highlights the complexity of microbiota-host interaction (26). Indole derivatives derived from gut microbiota can activate aryl hydrocarbon receptors (AhR), upregulate the expression of aquaporin 4 (AQP4) in astrocytes, and promote the clearance of β-amyloid protein (Aβ) (27). In contrast, the host’s metabolic shift toward the kynurenine pathway of tryptophan leads to the accumulation of quinolinic acid, which triggers oxidative stress through activation of NADPH oxidase (NOX2) and enhances the resistance of α-syn to proteasomal degradation (28). Mass spectrometry analysis has confirmed that the plasma quinolinic acid/tryptophan ratio in PD patients is positively correlated with iron deposition in the substantia nigra (evaluated by quantitative susceptibility mapping, QSM) (29). SCFAs and tryptophan metabolites jointly participate in the immune modulation and metabolic regulation of the central nervous system in PD, and the mechanistic basis will be further elaborated in the next section, “Vagal Connectivity Anatomy.”

### 2.2 Functional anatomy of gut-Nigral network connectivity

The vagus nerve, as the core communication pathway of the bidirectional gut-brain axis, projects from the dorsal motor nucleus (DMV) to the myenteric plexus of the gut, while its afferent fibers transmit intestinal mechanical/chemical signals to the nucleus tractus solitarius (NTS) via the nodose ganglion (30). Viral tracing experiments have confirmed that injection of pseudorabies virus (PRV) into the gut can retrogradely label the multisynaptic pathway from the NTS to the substantia nigra pars compacta (SNc) (31). Optogenetic studies have shown that specific activation of gut vagal C fibers can enhance theta rhythmic oscillations (4–8 Hz) of dopaminergic neurons in the SNc, thereby improving motor coordination (32). This mechanism may explain the clinical efficacy of transcutaneous vagus nerve stimulation (tVNS) in improving motor symptoms in PD patients (33).

### 2.3 Immuno-neural interaction of systemic inflammation

**Figure 1:**
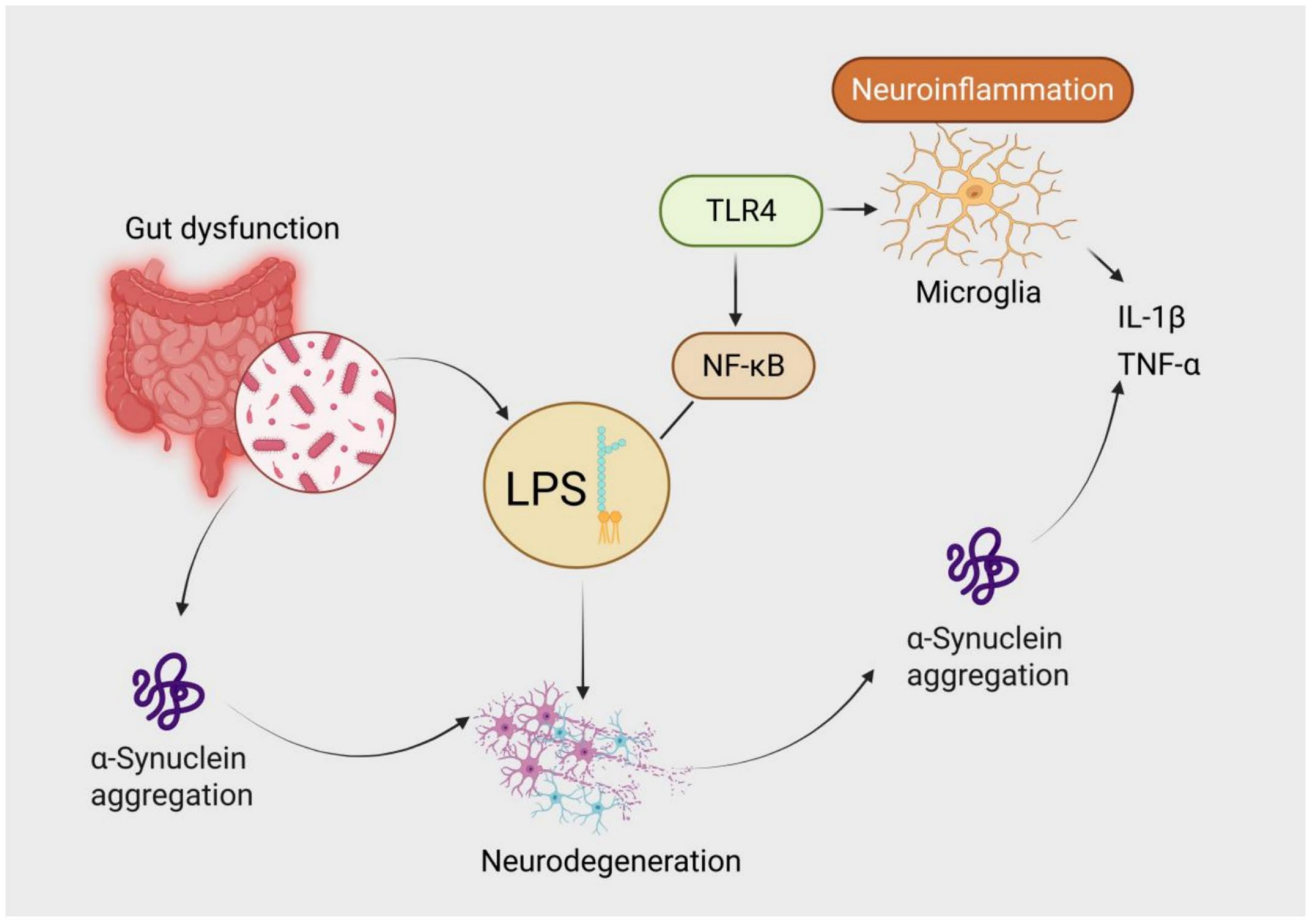
Mechanism of lipopolysaccharide (LPS)-mediated neuroinflammation in the gut-brain axis contributing to Parkinson’s disease pathogenesis. Gut dysfunction leads to dysbiosis and overgrowth of Gram-negative bacteria, resulting in elevated LPS translocation into systemic circulation. LPS activates Toll-like receptor 4 (TLR4) and triggers the NF-κB signaling cascade, leading to microglial activation and release of proinflammatory cytokines such as IL-1β and TNF-α. Both LPS and inflammation promote α-synuclein aggregation, ultimately driving neurodegeneration—a core pathological hallmark of Parkinson’s disease.

Destruction of intestinal barrier integrity leads to the translocation of pathogen-associated molecular patterns (PAMPs) such as lipopolysaccharide (LPS) into the bloodstream, aggravating central neurodegeneration via dual mechanisms: on the one hand, peripheral monocytes migrate to brain parenchyma via C-C chemokine receptor 2 (CCR2), differentiate into proinflammatory macrophages, and release IL-1β to activate microglial NLRP3 inflammasomes (34); on the other hand, LPS enhances the nucleation efficiency of α-syn fibrils via TLR4-dependent mechanisms, and the strength of this effect is positively correlated with the abundance of Gram-negative bacteria in the gut microbiota (35). Animal experiments have shown that knockout of TLR4 can significantly reduce dopaminergic neuron loss in the substantia nigra induced by α-syn preformed fibrils (PFFs) (36). Clinical neuroimaging evidence further supports this mechanism: serum LPS-binding protein (LBP) levels in PD patients are significantly correlated with the extent of microglial activation in the substantia nigra (quantified by ^11C-PK11195 PET) (37) (Figure 1).

### 2.4 Preclinical evidence linking the gut microbiome to PD

Multiple animal studies have demonstrated a causal relationship between gut microbiota alterations and Parkinsonian pathology. Sampson et al. showed that fecal microbiota transplants (FMT) from PD patients into germ-free mice exacerbated motor deficits and microglial activation compared to transplants from healthy controls, directly implicating gut dysbiosis in disease progression (19). In MPTP-induced PD mouse models, antibiotic treatment or SCFA supplementation modulated both motor behavior and neuroinflammation, indicating that gut-derived metabolites influence the central disease process (38). DSS-induced colitis, by disrupting the intestinal barrier and promoting systemic inflammation, has been shown to aggravate nigrostriatal neurodegeneration in PD-prone mice (39). Additionally, irradiation-based microbiota depletion followed by recolonization further confirmed that gut microbial composition can alter the severity of α-synuclein pathology and dopaminergic neuronal loss (40). These findings provide robust mechanistic support for a gut-origin component in PD pathogenesis.

## 3 Intervention strategies of remote rehabilitation on the gut-brain Axis

As the gut–brain axis becomes increasingly recognized as a therapeutic target in PD, a variety of strategies have emerged aiming to restore microbial homeostasis, reduce inflammation, and enhance barrier integrity. Table 1 provides a concise overview of the main pathological features associated with gut dysbiosis in PD and the interventions currently under investigation.

**Table 1:** Key gut microbiota-associated pathological changes in Parkinson’s disease and corresponding intervention strategies.

**Microbiome/metabolic abnormality**	**Pathological consequences**	**Key mechanisms**	**Potential interventions**
↓SCFA-producing bacteria	Energy metabolism impairment; barrier dysfunction	↓SCFAs → mitochondrial dysfunction; ↓tight junctions	Diet, prebiotics
↑LPS-producing Gram-negative bacteria	Neuroinflammation; α-syn aggregation	↑LPS → TLR4 → NF-κB → NLRP3 inflammasome → IL-1β	Probiotics, prebiotics, tVNS
↑Kynurenine pathway activation	Oxidative stress; mitochondrial toxicity	↑Quinolinic acid → NMDA receptor → Ca^2+^ overload;↑NOX2	Diet, probiotics, SCFA supplementation
Dysbiosis and reduced diversity	Gut-brain axis disruption	Altered microbial metabolites; loss of immune regulation	FMT, diet, synbiotics, tVNS
Gut barrier impairment	Systemic endotoxin leakage	↓mucus layer, ↑permeability → LPS translocation	Probiotics, polyphenols, dietary fiber

### 3.1 Neuromodulation interventions

To address vagus nerve signaling impairment and central circuit dysfunction, current studies have proposed non-pharmacological remote intervention strategies centered on neuromodulation. Digital therapeutics—software-driven tools that deliver therapeutic effects—provide a precise intervention path for gut-brain axis regulation (41). Biofeedback systems based on wearable devices dynamically assess autonomic nervous tone by continuously monitoring heart rate variability (HRV), allowing for real-time adjustment of the intensity and frequency of transcutaneous vagus nerve stimulation (tVNS) (42). Virtual reality (VR) technology reshapes neural plasticity through multi-sensory integration training. Functional near-infrared spectroscopy (fNIRS) shows that VR tasks induce gamma-band oscillations (30–80 Hz), which are preliminarily associated with improved synchronization of the substantia nigra–thalamus–cortex circuit and may facilitate motor initiation in PD (43). However, these findings remain primarily observational and require further validation in large-scale clinical trials. In animal models of neurodegenerative disease, multi-sensory stimulation interventions such as environmental enrichment, which refers to housing animals in a complex environment with toys, running wheels, and varied stimuli to promote sensory and motor engagement have demonstrated potential regulatory effects—e.g., increased striatal dopamine transporter (DAT) density in α-synuclein transgenic mice after 12 weeks of training. Whether such effects translate to human PD patients remains to be established (44). Based on these preclinical findings, VR technology is hypothesized to exert DAT-modulating effects in PD by mimicking visual–motor integration seen in enriched environments. However, its neuroprotective potential in humans remains speculative and warrants systematic clinical investigation.

### 3.2 Gut-targeted dietary interventions

Dietary interventions aim to modulate gut microbiota composition and metabolic capacity, potentially improving SCFA deficiency and LPS translocation. Personalized dietary programs are dynamically adjusted via mobile applications: low-FODMAP diets reduce the proliferation of gas-producing bacteria in the gut, thereby lowering serum lipopolysaccharide-binding protein (LBP) levels (45); meanwhile, the Mediterranean diet increases the abundance of *Akkermansia muciniphila*, promotes butyrate synthesis, and improves fecal microbiota alpha diversity (46).

### 3.3 Probiotic/prebiotic interventions

Combined probiotic and prebiotic interventions have shown potential for regulating gut-brain axis function through multiple mechanisms. Studies have shown that *Lactobacillus reuteri* DSM 17938 can improve gut microecology and exert anti-inflammatory effects. Intervention with *Lactobacillus reuteri* DSM 17938 reduces the abundance of potentially pro-inflammatory bacteria, such as hydrogen sulfide-producing species, thereby indirectly improving host mitochondrial function and metabolic status (47). Although the specific mechanisms remain under investigation, improvements in mitochondrial energy metabolism indicators (e.g., ATP levels) have been observed in animal experiments. A small-scale randomized controlled trial suggested that combined use of probiotics and prebiotics may increase brain-derived neurotrophic factor (BDNF) levels in cerebrospinal fluid (48). These findings are preliminary and require replication in larger cohorts. A meta-analysis reported associative improvements in gastrointestinal symptoms following combined probiotic–prebiotic supplementation in PD patients, with reductions in symptom scores and inflammatory markers such as serum IL-6 (49). However, due to heterogeneity in included studies and modest effect sizes, causal conclusions remain tentative. These findings suggest that combined probiotic-prebiotic interventions may simultaneously target gut microbiota metabolism, intestinal barrier protection, and systemic inflammation alleviation, holding broad prospects for application.

## 4 Multimodal biomarkers and efficacy evaluation

### 4.1 Integrated analysis of Fecal metabolomics and neuroimaging

Fecal metabolomics analyses have revealed systematic disturbances in gut-derived metabolites in PD patients, particularly the reduction of short-chain fatty acids (SCFAs) such as butyrate, which is closely related to disease pathogenesis (50–52). Yang, X et al. (53) found that the concentrations of butyrate, propionate, and acetate were significantly reduced in the feces of PD patients, which may impair gut barrier function and influence neuroinflammatory states. Resting-state functional magnetic resonance imaging (rs-fMRI) studies have shown that the functional connectivity of the default mode network (DMN) is closely associated with cognitive performance in PD patients (54). Decreased DMN connectivity in PD patients correlates with cognitive impairment (55). Although direct studies linking fecal metabolomic data to neuroimaging indicators (e.g., nigral iron deposition) are currently lacking, the above findings suggest that changes in gut metabolic profiles may influence brain network function and participate in PD pathogenesis.

### 4.2 Integrated application of clinical assessment tools

With the continuous development of multimodal intervention strategies, a corresponding system of integrated evaluation indicators is gradually being established to enable closed-loop analysis among mechanisms, symptoms, and interventions. The Movement Disorder Society-Unified Parkinson’s Disease Rating Scale (MDS-UPDRS) is the standard tool for assessing both motor and non-motor symptoms in PD patients (56–58). Biomarkers of intestinal permeability in PD, such as elevated serum zonulin levels, are positively correlated with MDS-UPDRS scores, suggesting that gut barrier dysfunction may be associated with the severity of motor symptoms (50). In terms of motor function assessment, wearable inertial sensors have been used to capture gait features in PD patients (59–62). One study employed machine learning algorithms such as XGBoost to analyze gait data and successfully distinguished PD patients from healthy controls with high accuracy (63). Although this study did not directly associate gait analysis with gut microbiota diversity, it indicates that the integration of multimodal data may contribute to individualized evaluation of PD. However, current assessment methods still face many challenges in longitudinal tracking, causal inference, and cross-center standardization, requiring further refinement in future research.

## 5 Challenges and future directions

### 5.1 Limitations of mechanistic studies

Although a large number of studies have elucidated the pathogenesis of PD from classical pathways such as α-synuclein aggregation, most of these findings are derived from animal models, which differ substantially from the actual human disease course (3, 64). In animal models, bilateral loss of dopaminergic neurons in the substantia nigra pars compacta (SNpc) can be observed 6 months after striatal injection of α-synuclein preformed fibrils (PFFs), with α-synuclein inclusions forming within 2 months; the extent of neuronal loss is dose-dependent on the injected PFFs (65). However, in clinical practice, PD is typically diagnosed when neuronal loss has already reached 60 to 80%, suggesting that interventions in the prodromal phase may be more effective (66). This discrepancy underscores a major translational gap: animal models often rely on rapid-onset, artificially induced PD phenotypes that do not fully recapitulate the gradual, multifactorial progression seen in humans. Moreover, many preclinical interventions showing promise in rodents have failed to replicate efficacy in clinical trials, reflecting fundamental differences in pathophysiology and treatment response.

Inter-individual genetic differences also influence the efficacy of interventions. For example, PINK1 and Parkin genes play essential roles in maintaining mitochondrial quality control, and their mutations are closely associated with PD onset (67). These findings underscore the importance of integrating multi-omics data to develop personalized therapeutic strategies.

### 5.2 Translational bottlenecks in technology

As remote monitoring and data collection gradually enter clinical practice, sample processing and behavioral adherence assessment have become key technical bottlenecks in the implementation of intervention strategies. In remote biosample collection, storage conditions significantly affect metabolite stability (68). Studies have shown that the concentration of butyrate in fecal samples decreases by 37% after 24 h at room temperature but only by 8% when stored at 4 °C (69). Differences in sampling tools may also cause deviations in microbial abundance detection, affecting the accuracy of research results (70). Beyond sampling inconsistencies, another critical bottleneck lies in the validation and regulatory approval of digital biomarkers derived from wearable data. Many machine learning-based models lack external validation and suffer from limited generalizability across populations. Moreover, regulatory frameworks for integrating microbiome-based or digital readouts into clinical decision-making are still in their infancy, delaying real-world implementation.

In adherence assessment for digital therapeutics, traditional questionnaire-based methods are often subjective and error-prone (71, 72). The introduction of blockchain technology (e.g., Hyperledger Fabric framework) enables real-time encrypted recording of data, enhancing data reliability and transparency (73). Baucas et al. (74) proposed a fog computing–IoT platform integrating federated learning and blockchain for wearable device applications in predictive medicine. This platform aims to ensure the security of patient data and the integrity of predictive services through a distributed structure and privacy protection mechanisms. The results showed that the proposed implementation effectively protects patient privacy and maintains the integrity of predictive services (74).

Wearable sensors combined with machine learning algorithms have shown promising potential in the diagnosis and severity assessment of PD though their integration with microbiome-based monitoring remains an emerging field under active development (75–77). One study used wearable sensors to collect gait data, and applied XGBoost algorithms to successfully distinguish PD patients from healthy controls with high accuracy (78).

### 5.3 Exploration of cutting-edge cross-disciplinary fields

The relationship between the gut microbiota and PD is receiving increasing attention (79–81). Krueger et al. (82) found that the abundance of butyrate-producing bacteria was reduced in the gut of PD patients, leading to lower butyrate levels, which may affect gut barrier function and neuroinflammatory status. The signaling mechanisms between the gut and brain are also being progressively revealed (83, 84). Vitetta et al. reported that neuroepithelial cells in the gut can rapidly transmit signals to the brain via the vagus nerve, influencing neuronal activity (85). Optogenetic technology provides new tools for studying the gut–brain axis (86, 87). By specifically activating light-sensitive channels in intestinal enterochromaffin (EC) cells, serotonin (5-HT) release can be modulated, thereby affecting central nervous system function (88). These emerging technologies offer unprecedented methodological support for elucidating peripheral–central signaling coupling mechanisms and developing non-invasive intervention approaches, which warrant systematic integration and validation in future studies.

### 5.4 Conclusion

**Figure 2:**
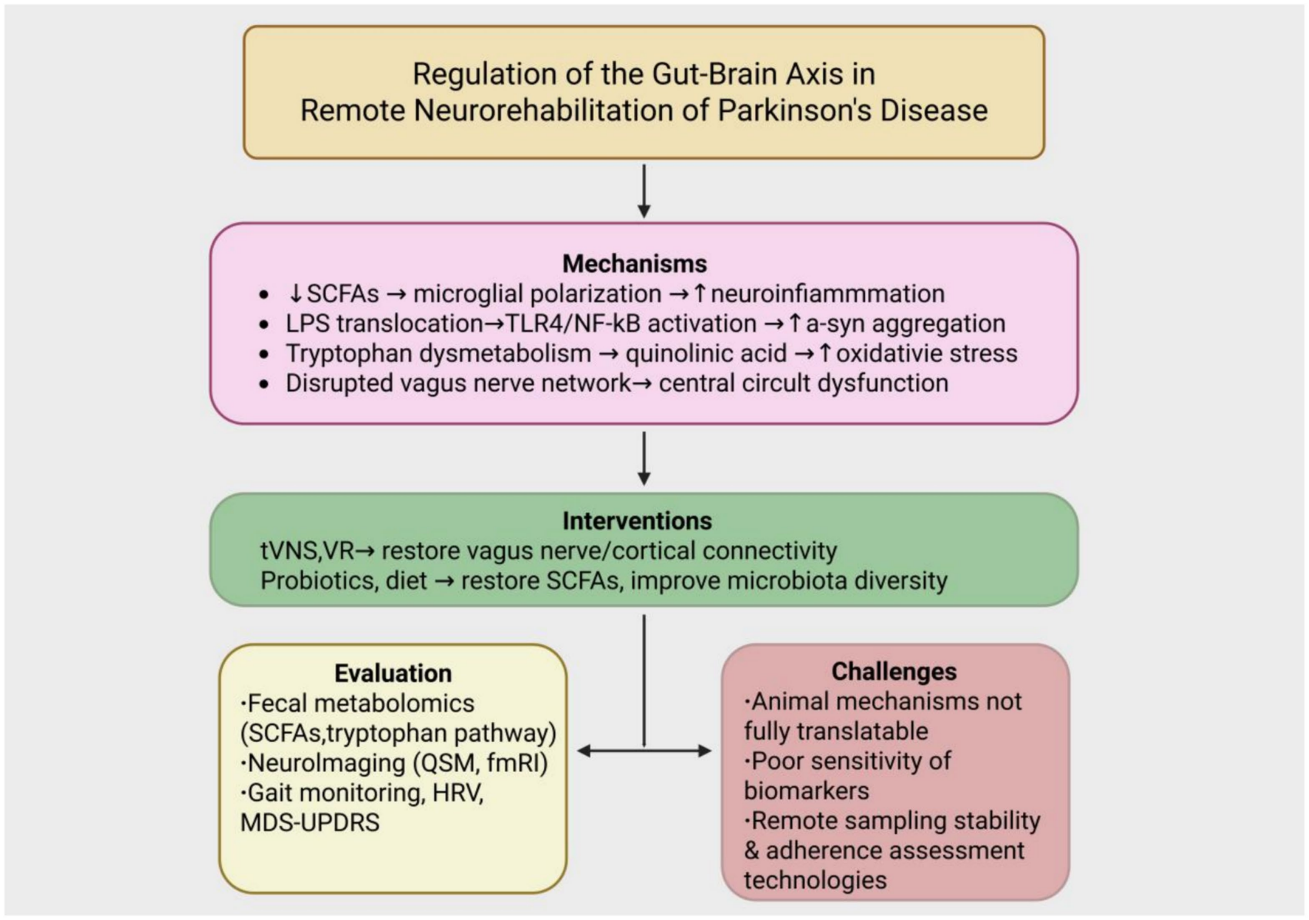
Integrated framework of gut–brain axis regulation in remote neurorehabilitation of Parkinson’s disease. The diagram illustrates the pathological mechanisms of PD involving reduced short-chain fatty acids (SCFAs), LPS translocation, tryptophan dysmetabolism, and vagus nerve dysfunction. These are targeted by non-pharmacological interventions such as transcutaneous vagus nerve stimulation (tVNS), virtual reality (VR), and gut microbiota modulation (probiotics and diet). Multimodal tools for evaluation include fecal metabolomics, neuroimaging, and gait/HRV monitoring. Key translational challenges include limited clinical applicability of animal models, low biomarker sensitivity, and inadequate remote adherence tracking systems.

The pathogenesis of Parkinson’s disease is gradually expanding from the traditional concept of central nervous system degeneration to the interplay between the gut and brain axis (as illustrated in Figure 2).

Current studies suggest that factors such as gut microbiota imbalance, abnormal metabolite profiles, and impaired gut barrier function may play roles as early as the prodromal phase of the disease. These factors contribute to neuronal injury via multiple mechanisms, including neuroinflammation, mitochondrial dysfunction, and α-synuclein aggregation. The vagus nerve, as a key pathway of the gut–brain axis, not only mediates signal transmission under physiological conditions but may also act as a conduit for pathological protein propagation from the periphery to the brain under pathological conditions.

In terms of intervention strategies, remote rehabilitation techniques such as transcutaneous vagus nerve stimulation, virtual reality training, and wearable device monitoring offer new tools for personalized management of Parkinson’s disease. Meanwhile, gut-targeted approaches including probiotics, prebiotics, and dietary interventions have shown potential in preclinical and early clinical studies to modulate gut microbiota, attenuate systemic inflammation, and support gut barrier function; however, their efficacy and mechanisms in PD patients remain to be fully validated. Integrating fecal metabolomics, brain imaging, motor parameters, and immune markers into a multimodal framework may support early detection and treatment monitoring. The envisioned integration of microbiome biomarker analytics with wearable technologies represents a promising but as yet unrealized frontier in precision remote management of PD. While the convergence of omics technologies, wearable devices, and digital therapeutics holds transformative promise, substantial translational hurdles remain. These include the poor replicability of animal-derived findings in heterogeneous human populations, the lack of standardized pipelines for digital biomarker validation, and the limited regulatory infrastructure to support clinical adoption.

Despite current limitations such as unclear mechanisms, lack of sensitive biomarkers, and inadequate remote data collection and adherence assessment tools, ongoing advances in biotechnology, artificial intelligence, and interdisciplinary integration are expected to further advance gut–brain axis research and intervention strategies for Parkinson’s disease. These developments will provide a more solid foundation for early diagnosis and comprehensive treatment.
